# Noninvasive method to estimate anaerobic threshold in individuals with type 2 diabetes

**DOI:** 10.1186/1758-5996-3-1

**Published:** 2011-01-12

**Authors:** Marcelo M Sales, Carmen Sílvia G Campbell, Pâmella K Morais, Carlos Ernesto, Lúcio F Soares-Caldeira, Paulo Russo, Daisy F Motta, Sérgio R Moreira, Fábio Y Nakamura, Herbert G Simões

**Affiliations:** 1 Graduate program in Physical Education of the Catholic University of Brasilia, Águas Claras, Taguatinga-DF, 72022-900, Brazil; 2 Physical Education Faculty of the North University of Parana, Paris avenue, 675, Jardim Piza - Londrina - Paraná, 86041-120, Brazil; 3 Physical Education Faculty of the Federal Institute of Roraima, Capitão Júlio Bezerra avenue, 1392, Aparecida, Boa Vista - Roraíma, 69303-340, Brazil; 4 Departament of Physiology and Biophysics - Federal University of Minas Gerais, Antônio Carlos avenue, 6627, Pampulha, Belo Horizonte - Minas Gerais, 31270-901, Brazil; 5 Physical Education Faculty of the Federal University of Vale do São Francisco, José de Sá Maniçoba avenue, Center, Petrolina - Pernambuco, 56304-205, Brazil; 6 Graduate program in Physical Education of the State University of Londrina, Celso Garcia Cid highway, 380 kilometer, Londrina - Paraná, 86051-980, Brazil

## Abstract

**Background:**

While several studies have identified the anaerobic threshold (AT) through the responses of blood lactate, ventilation and blood glucose others have suggested the response of the heart rate variability (HRV) as a method to identify the AT in young healthy individuals. However, the validity of HRV in estimating the lactate threshold (LT) and ventilatory threshold (VT) for individuals with type 2 diabetes (T2D) has not been investigated yet.

**Aim:**

To analyze the possibility of identifying the heart rate variability threshold (HRVT) by considering the responses of parasympathetic indicators during incremental exercise test in type 2 diabetics subjects (T2D) and non diabetics individuals (ND).

**Methods:**

Nine T2D (55.6 ± 5.7 years, 83.4 ± 26.6 kg, 30.9 ± 5.2 kg.m^2(-1)^) and ten ND (50.8 ± 5.1 years, 76.2 ± 14.3 kg, 26.5 ± 3.8 kg.m^2(-1)^) underwent to an incremental exercise test (IT) on a cycle ergometer. Heart rate (HR), rate of perceived exertion (RPE), blood lactate and expired gas concentrations were measured at the end of each stage. HRVT was identified through the responses of root mean square successive difference between adjacent R-R intervals (RMSSD) and standard deviation of instantaneous beat-to-beat R-R interval variability (SD1) by considering the last 60 s of each incremental stage, and were known as HRVT by RMSSD and SD1 (HRVT-RMSSD and HRVT-SD1), respectively.

**Results:**

No differences were observed within groups for the exercise intensities corresponding to LT, VT, HRVT-RMSSD and HHVT-SD1. Furthermore, a strong relationship were verified among the studied parameters both for T2D (r = 0.68 to 0.87) and ND (r = 0.91 to 0.98) and the Bland & Altman technique confirmed the agreement among them.

**Conclusion:**

The HRVT identification by the proposed autonomic indicators (SD1 and RMSSD) were demonstrated to be valid to estimate the LT and VT for both T2D and ND.

## Introduction

The maximal oxygen consumption (VO_2max_) and anaerobic threshold (AT) are parameters that have been widely considered as hallmarks of aerobic fitness both for athletes [1], physically active individuals [2] and, in a minor scale, for special populations such as individuals with type 2 diabetes (T2D) [3,4].

Regular exercise provides many physiologic benefits, reduces risk of disease outcomes, and triggers important psychological gains in healthy and pathological conditions. Moreover, few studies have evaluated the effect of different exercise intensities on T2D [5]. The AT can be used in this specific evaluation and has been considered a gold standard parameter for exercise prescription including cardiovascular risks groups [3,6].

While several studies have identified the AT through the responses of blood lactate [2,7,8] ventilation [9] and blood glucose[7] others have suggested the response of the heart rate variability (HRV) as a method to identify the AT in young healthy individuals [10]. The HRV is a non-invasive measure of the oscillation between consecutive cardiac cycles (as measured between each R-R complex). This technique has been used for the noninvasive evaluation of the sympathetic (SNS) and parasympathetic nervous system (PNS) activity both during resting and low intensity exercise[11].

During low to moderate exercise intensity the heart rate (HR) increase is mainly controlled by the PNS withdrawal [12]. On the other hand, at higher intensities (e.g. above AT), there is a reduction in the parasympathetic modulation concomitant to an increase in sympathetic activity [13,14] and thus a decrease in HRV is observed [15]. Such transition between the increase in the SNS activity and the vagal tone withdrawal occurs close to 60% VO_2max_, being close to the intensity at which the lactate (LT) and ventilatory (VT) thresholds have been observed for healthy young individuals [10,15]. However, the validity of the HRV in estimating the LT and VT for individuals who may have autonomic dysfunction associated with T2D [16-18] has not been previously examined.

The HRV as a measure to estimate the AT is noninvasive and inexpensive, providing information regarding the autonomic regulation during exercise. In spite of presenting a higher sympathetic activity during resting and light exercise [19,20] the HRV responses of T2D is thought to be sensitive to predict the LT and VT noninvasively by considering the intensity at which the vagal withdrawal occurs. However, the validity of HRV in estimating the LT and VT for individuals with T2D has not been investigated yet.

Thus, the purposes of present study were 1) to analyze the possibility of identifying the heart rate variability threshold (HRVT) by considering the responses of parasympathetic indicators during incremental exercise test, and 2) to compare and verify the relationships between the HRVT with the VT and LT for individuals with (TD2) and without type-2 diabetes (ND).

## Methods

### Participants

After approval of the local Ethics Committee for Human Research and obtaining written informed consent of the volunteers, 9 T2D individuals, being 4 male and 5 female (55.6 ± 5.7 years, 83.4 ± 23.6 kg, 30.9 ± 5.2 kg.m^2(-1)^) and 10 ND, being 8 male and 2 female (50.8 ± 5.1 years, 76.2 ± 14.3 kg, 26.5 ± 3.8 kg.m^2(-1)^) participated in this study. Diabetes diagnostic confirmation was conducted through previous medical screening and laboratory measurements such as blood glucose and estimates of glycated hemoglobin (HbA_1c_)[21]. The individuals with T2D were on medical and nutritional treatment, using oral hypoglycemiants (*Sulfonylureas, Metformin, Metformin+Glibenclamide, Glimepiride, Pioglitazone Chloridrate*) and/or controlled food intake. Exclusion criteria for participation in this study included previous diagnosis of peripheral and autonomic neuropathy, retinopathy, as well as diabetic foot ulcerations and orthopedic complications, the use of insulin, and/or use of medicines that would directly affect the HR, or even any other problem that would impair the participants' ability to complete all the study procedures. The general characteristics of the volunteers are presented in table 1.

**Table 1:** Characteristics of the type 2 diabetics individuals (T2D) (n = 9) and non diabetics individuals (ND) (n = 10).

	T2D	ND
**Age (years)**	55.6 ± 5.7	50.8 ± 5.1
**Weight (kg)**	83.4 ± 23.6	76.2 ± 14.3
**Height (cm)**	163.1 ± 10.6	169.0 ± 9.2
**BMI (kg.m^2(-1)^)**	30.9 ± 5.2	26.5 ± 3.8
**Time of disease (years)**	6.0 ± 2.3	-
**Fasting blood glucose (mg.dL^-1^)**	138.6 ± 34.2*	91.1 ± 5.5
**HbA_1c _(%)**	6.8 ± 1.3*	5.2 ± 0,4
**VO_2peak _(mL.kg.^-1^.min^-1^)**	20.6 ± 4.3*	29.4 ± 6.8
**RMSSD_rest _(ms)**	21.2 ± 3.5	26.3 ± 5.1
**SD1_rest _(ms)**	15.3 ± 2.5	20.9 ± 4.6

Data expressed as mean (± standard deviation) and standard error of mean for heart rate variability variables (RMSSD and SD1).

BMI = body mass index; VO_2peak_= peak oxygen uptake; HbA_1c_= glycated hemoglobin estimated as suggested Rohlfing et al.[21]; RMSSD_rest_= square root of the mean squared successive differences between adjacent R-R intervals in rest; SD1_rest_= standard deviation of variability instantaneous beat-to-beat in rest. *p < 0.05 in relationship to ND group.

### General procedures

All experimental sessions were carried out in the Laboratory of Physical Evaluation and Training (LAFIT) of the Catholic University of Brasília, during the morning period, 2 h after volunteers had ingested a standardized breakfast consisting of 315.9 kcal as follows: 53 g (67.1% - 212 kcal) of carbohydrate; 4.6 g (5.8%- 18.3 kcal) of protein and 9.5 g (27.1%-85.6 kcal) of fat, which was considered a moderate glycemic index meal (GI = 73.9). The participants were submitted to a physician evaluation, including resting electrocardiogram (ECG) (ELITE, Micromed^®^) and blood pressure (BP) measurements (BP 3AC1-1 Microlife Co.), besides anthropometric measurements and incremental exercise test. During the incremental exercise test, measurements of HR, BP, rate of perceived exertion (RPE), as well as ventilatory and blood lactate variables were continuously monitored.

### Incremental exercise test and measurements

The incremental test (IT) was performed on an electromagnetic cycle ergometer (*Lode Excalibur Sport - Netherlands*) with 15 watts of initial workload and 15 watts increments at each 3-min stage until volitional exhaustion, maintaining 60 revolutions per minute. The cardiologist of the laboratory monitored the ECG of the volunteers throughout the test in order to identify any possible abnormality during exercise.

During the pre-exercise resting, as well as during the last 20 s of each incremental stage, a 25 μL sample of capillarized blood was collected from the ear lobe trough heparinized and calibrated glass capillaries, being deposited in microtubes (*Eppendorf*) containing 50 μL of sodium fluoride (NaF) 1% for latter analyses of the [Lac] through an electroenzimatic method (Yellow Springs 2.700 STAT, OH, USA).

Expired gases were also measured during the IT through a gas analyzer (Metalyzer 3B, Cortex Biophysik, Germany) that was previously calibrated with a 3-L syringe (flow calibration) and a standard mixed gas containing 4.9% CO_2 _and 17% O_2 _(gas calibration). The values of ventilation (VE), oxygen uptake (VO_2_) and carbon dioxide production (VCO_2_) were recorded during the last 20 s of each 3-min stage.

During the entire IT, the R-R intervals were recorded (Polar^® ^S810i, Polar Electo Oy, Kempele, Finland) [22] and filtered in the Polar Precision Performance (v. 4.0) software.

### Lactate threshold (LT) determination

The blood lactate kinetics during the IT stages identified the LT, being considered an exercise intensity above which an over proportional increase in blood lactate was observed in relation to increasing workload [1].

### Ventilatory threshold (VT) and VO_2peak _determination

Expired gases were collected breath by breath and the data from the last 20 seconds. The VT was determined through the analyses of the ventilatory equivalents of O_2 _(VE/VO_2_) and CO_2 _(VE/VCO_2_), being considered the intensity corresponding to the moment that VE/VO_2 _presented an over proportional increase in relation to VE/VCO_2_[23]. VO_2peak _was considered as the highest oxygen consumption attained at the exhaustion moment [24].

### Heart rate variability threshold determination (HRVT)

Initially, the R-R intervals were analyzed by the time domain through the square root of the mean squared successive differences between adjacent R-R intervals (RMSSD). Also, heart rate variability (HRV) was analyzed through the Poincaré plotting technique, from which the instantaneous variability of beat to beat data was derived by means of SD1, according to procedures previously described [15,25]

The RMSSD and SD1 corresponded to the R-R interval measures of the last 60 s of each 3-min stage during the IT. All analyses were run through the HRV Analysis v1.1 software (Biosignal Laboratory, University of Kuopio, Finland).

**Figure 1:**
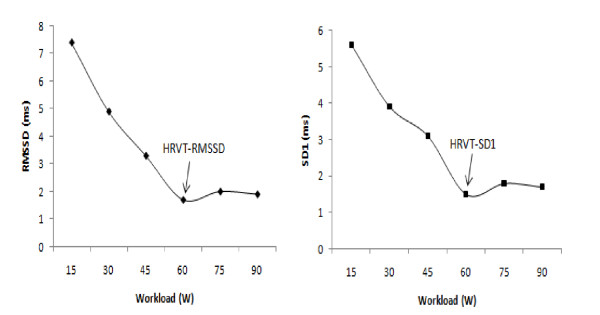
**Determination of the heart rate variability threshold (HRVT-RMSSD and HRVT-SD1), for type 2 diabetes mellitus individuals**.

For the determination of the HRVT, a stabilization point lower than 3 milliseconds (ms) was adopted for of the vagal activity indices (SD1 and RMSSD) plotted against the absolute workload (Figure 1) [11,25,26]

### Data Analysis

The data are presented as means (± SD). In order to compare the characteristics of the groups, the Student-t test for independent samples was applied. After assessing normality of data through the test of Skewnees and Kurotsis, comparisons between and within groups were done by using an ANOVA for repeated measures, with *Tukey test *as *post hoc*. The Pearson's product moment correlation was used to verify the relationships among the studied methods to determine AT and the Bland & Altman technique was applied to verify the agreement among different methods [11,27]. In addition, we also applied the kappa index between the methods of identifying the anaerobic threshold. The significance level adopted was *P *< 0.05 (*Statistica^® ^version 5.0*).

## Results

The results presented in table 2 show no significant differences among the workloads (W) corresponding to AT identified by different methods both groups. For both groups the HR, [Lac], VO_2 _and RPE corresponding to the AT identified by different techniques did not differ from each other (Table 2). Further, for the T2D group significant correlations (p < 0.01) between LT and peak power (W_peak_) (r = 0.89), VT and W_peak _(r = 0.75), HRVT-RMSSD and W_peak _(r = 0.80) and between HRVT-SD1 and W_peak _(r = 0.80) were observed. High correlations (p < 0.001) were also found in the ND group for the LT and W_peak _(r = 0.95), VT and W_peak _(r = 0.92) and HRVT-RMSSD and W_peak _(r = 0.89) and between HRVT-SD1 (r = 0.90). The relationship among exercise intensities (W) corresponding to the studied thresholds are presented in table 3 for both groups.

**Table 2:** Mean (± SD) of workload, blood lactate, heart rate and the rate of perceived exertion corresponding to the lactate (LT), ventilatory (VT) and heart rate variability thresholds (HRVT-RMSSD and HRVT-SD1).

	Parameter	Worload (W)	VO_2 _(mL.kg.^-1^min^-1^)	[LAC] (mmol.L^-1^)	HR (bpm)	RPE (score)
**T2D**	**LT**	55.0 ± 26.0	13.0 ± 1.2	2.9 ± 0.6	126.4 ± 14.1	13.0 ± 2.1
	**LV**	58.3 ± 23.0	13.3 ± 1.8	3.0 ± 1.0	128.8 ± 14.4	13.2 ± 2.0
	**HRVT-RMSSD**	58.3 ± 17.5	13.3 ± 1.1	3.0 ± 0.7	128.2 ± 8.9	13.6 ± 1.4
	**HRVT-SD1**	50.0 ± 15.0	13.0 ± 2.1	2.8 ± 0.9	123.2 ± 6.9	12.9 ± 1.8
	**Exhaustion**	96.7 ± 33.6‡	20.6 ± 4.6‡	6.5 ± 2.5‡	159.7 ± 20.8‡	18.4 ± 1.6‡
						
**ND**	**LT**	85.5 ± 33.2*	19.2 ± 5.9*	3.6 ± 0.6*	139.3 ± 17.4	14.3 ± 1.4
	**LV**	91.5 ± 28.7*	20.3 ± 5.6*	4.1 ± 1.1*	143.1 ± 16.2	14.6 ± 1.2
	**HRVT-RMSSD**	85.5 ± 34.7*	19.0 ± 5.3*	3.5 ± 0.7	137.7 ± 13.1	13.9 ± 1.2
	**HRVT-SD1**	82.5 ± 34.1*	18.6 ± 5.3*	3.2 ± 0.7	131.2 ± 13.8	13.7 ± 1.2
	**Exhaustion**	135.0 ± 45.3‡	29.4 ± 6.8‡	7,7 ± 2.0‡	173.6 ± 9.3‡	18.8 ± 1.3‡

Exhaustion in T2D (n = 9) and ND (n = 10).

T2D = individuals with type 2 diabetes; ND = individuals without type-2 diabetes; *p < 0.05 in relation to corresponding results for T2D; ‡ p < 0.05 in relation to the thresholds (LT, TV, HRVT RMSSD and HRVT-SD1). No differences were observed within groups for the methods to identify anaerobic threshold intensities.

**Table 3:** Relationship among intensities (W) corresponding to the studied thresholds for T2D (n = 9) and ND (n = 10).

		LT (W)	VT (W)	HRVT-RMSSD (W)
**T2D**	**VT (W)**	0.83*	-	-
	**HRVT-RMSSD(W)**	0.85*	0.83*	-
	**HRVT-SD1 (W)**	0.87*	0.76*	0.68*
				
**ND**	**VT (W)**	0.95*	-	-
	**HRVT-RMSSD (W)**	0.94*	0.91*	-
	**HRVT-SD1 (W)**	0.96*	0.93*	0.98*

T2D = Type 2 diabetes mellitus individuals; ND = non diabetics individuals; LT = Lactate threshold; VT = ventilatory threshold; HRVT-RMSSD = heart rate variability threshold for RMSSD; HRVT-SD1 = heart rate variability threshold for SD1. *significant correlation p < 0.01.

**Figure 2:**
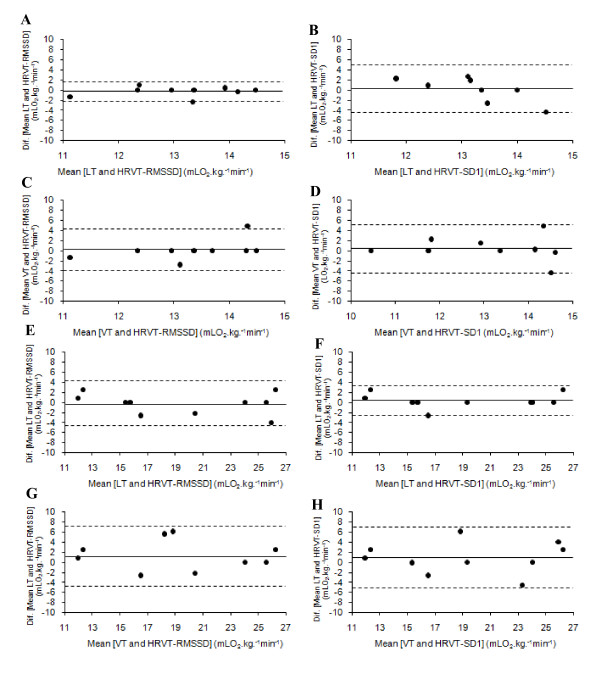
**Agreement between VO_2 _corresponding to LT, VT and HRVT-RMSSD and HRVT-SD1 for type 2 diabetes mellitus individuals (2 A, B, C, D) and non diabetes individuals (2 E, F, G, H)**.

The Bland and Altman plots showed good agreement for the VO_2 _corresponding to LT, VT, HRVT-RMSSD and HRVT-SD1 based on the low bias and relatively narrow limits of agreement [Bias (± 95% of confidence interval)] presented in both groups (T2D and ND) (Table 4 and Figure 2). In addition, the Kappa index also evidenced a good to excellent agreement among studied parameters for both T2D (K = 0.66 to K = 0.92) and ND (K = 0.62 to K = 0.92).

**Table 4:** Agreement between VO_2 _corresponding to LT, VT and HRVT-RMSSD and HRVT-SD1 for type 2 diabetes mellitus individuals (n = 9) and non diabetes individuals (n = 10).

Groups	Reference methods	Testing methods	Mean of differences	Limits of Agreement
**T2D**	**LT**	**HRVT-RMSSD**	0.3	± 2.0
	**LT**	**HRVT-SD1**	0.1	± 4.6
	**VT**	**HRVT-RMSSD**	0.1	± 4.1
	**VT**	**HRVT-SD1**	0.5	± 4.9
				
**ND**	**LT**	**HRVT-RMSSD**	-0.3	± 4.2
	**LT**	**HRVT-SD1**	0.3	± 2.9
	**VT**	**HRVT-RMSSD**	1.3	± 5.9
	**VT**	**HRVT-SD1**	0.9	± 6.2

T2D = Type 2 diabetes individuals; ND = non diabetes individuals; LT = lactate threshold; VT = ventilatory threshold; HRVT-RMSSD = Heart rate variability threshold for RMSSD; HRVT-SD1 = Heart rate variability threshold for SD1.

## Discussion

The ability of the HRV responses in estimating LT and VT was investigated in individuals with T2D and ND. The findings confirmed our hypothesis that AT could be identified in T2D through the parasympathetic markers of the HRV responses similarly to ND, being not different and highly correlated to the LT and VT for both groups (Tables 2 and 3, Figure 2). The HRV prediction of AT through autonomic variables such as RMSSD and SD1 has been studied in different populations [10,15,28,29]. However, this is the first study in which the AT was identified through HRV responses in T2D, in spite of the fact that this disease is associated with autonomic dysfunction [30,31].

The technique of HRV, as used in the present study, considered a progressive reduction of RMSSD and SD1 to intervals lower than 3 ms as markers of AT intensity as proposed by others [10,26]. Lima and Kiss [26] found a high relationship between workloads (Watts) corresponding to HRVT and LT for healthy subjects (r = 0.76). In this study, even higher correlations were observed between LT and RMSSD threshold (r = 0.85 and r = 0.94) and between LT and SD1 threshold (r = 0.87 and r = 0.96) for T2D and ND groups, respectively.

The main findings of present study evidenced the HRV as an effective parameter for exercise evaluation in individuals with T2D, due to its noninvasive characteristic as well as because it evaluates variables associated with autonomic function. According to Yeckel et al. [30] the autonomic dysfunction usually precedes the occurrence of T2D. In addition, an elevated sympathetic activity is associated to a higher risk for cardiovascular events such as infarction and stroke [32,33]. The HRVT seems to identify an exercise intensity that represents the transition from a lower to a higher sympathetic activity and thus to an increased probability for the occurrence of cardiovascular events T2D individuals. Therefore, the identification of the HRVT intensity may be a safe and useful procedure for exercise evaluation and prescription.

Another benefit of HRVT identification is the avoidance of elevated financial costs usually associated with invasive methods. Exercise tests involving blood collection and biochemical measures, and/or the use of expensive apparatus for expired gases analysis, as well as trained professionals dealing with such protocols are expensive and thus of difficult access to a major part of the population.

It has been suggested that vagal withdraw progressively occurs during IT, and is almost completely inhibited at intensities around 50-60% VO_2max_[16,29]. For the T2D participants of the present investigation, the vagal withdrawal, as observed by the RMSSD and SD1 stabilization, occurred at intensities corresponding to 64.8% and 66.6% of VO_2peak_, respectively. Previous studies with T2D also verified similar results regarding LT occurring at intensities around 66% VO_2peak _[4,34,35] what, in turn, may be of practical application.

The fact that participants of the present study had their HRVT being reached at 64.2 to 66.6% VO_2peak _may be at least partially explained by their low physical fitness (VO_2peak _of 20.6 ± 4.3 mL.kg^-1^.min^-1 ^for T2D and 29.4 ± 6.8 mL.kg^-1^.min^-1 ^for ND). This finding was also observed in a previous study comparing sedentary T2D individuals to physically active T2D and ND counter partners [35]. Moreira et al. also observed a trend towards lower [Lac] during the IT for T2D group, suggesting lower motor units recruitment and thus lower anaerobic glycolytic activity, lower power output, blood lactate and VO_2 _at the exhaustion moment as compared to ND, similar to what happened in present study [34].

The RPE may also be an interesting noninvasive tool for exercise evaluation and prescription for both T2D and ND. The RPE integrates cardiovascular, respiratory, neuromuscular and metabolic inputs as a result of increasing exercise intensity [36]. Some studies have demonstrated that during incremental exercise test, RPE scores around 12-13, as measured through the 15-point Borg scale, is related to attainment of the AT [2,9]. It was observed that RPE scores corresponding to AT in present study were around 12.9 ± 1.8 to 13.6 ± 1.4 for T2D, and 13.7 ± 1.2 to 14.6 ± 1.2 for ND (Table 2). The use of RPE for controlling exercise intensities may be an useful tool to ensure exercise prescription for T2D, once the RPE of 12-14 may help to delimitate the transition of moderate exercise to exercise intensities which eliciting blood glucose to increase [2,8,37,38].

One of the limitations of present study was that the maximal lactate steady state (MLSS) intensity was not determined for comparison to the studied protocols. The MLSS determination is considered the gold standard method among those of exercise evaluation through blood lactate response to exercise. Denadai et al. [39] and Van Schuylenbergh et al. [40] have shown this parameter to be highly correlated and not different to the LT and VT intensities. So we may suggest that the methods adopted on present study (LT and VT determinations), which did not differ from HRVT, would also represent the MLSS intensity.

Several investigations have shown a relationship between the exercise intensity at which the vagal withdrawn is observed with those corresponding to VT [15,41] and LT [10,26]. In the present study the HRVT, as identified through the quantitative analyses of the Poincaré plots (SD1), occurred at exercise intensities not different to the LT and VT. On the other hand, in spite of non-detectable statistical differences, the exercise intensities associated to the occurrence of the HRVT-SD1 trend to be slightly lower than LT and VT for both T2D and ND, but without statistical difference (p > 0.05). Similarly to our findings, Shibata et al. [42] observed that HR corresponding to the HRVT was lower than VT, and the authors suggested that vagal withdrawal would precede the VT occurrence during IT. It is expected that mechanisms responsible for homeostasis during exercise may be related to the occurrence of both VT and LT, and is also dependent to the sympathetic/parasympathetic balance. Thus, it is reasonable that the HRVT occurs at exercise intensities closely related to LT and VT intensities. Thus, once the HRVT identified by the RMSSD seemed to better predicts the LT and VT as evidenced by the Bland and Altman analysis, we may suggest that RMSSD may be a more accurate variable and thus should be preferred in relation to SD1.

In conclusion it was possible to identify the HRVT from autonomic indices (RMSSD and SD1) for both T2D and ND, with no differences in relation to LT and VT. Furthermore, high correlations and good agreement between the examined methods were observed, suggesting the validity of HRVT even for T2D. Additional studies are necessary to investigate the reproducibility and sensitivity of AT as identified by HRV measures to various interventions, including physical training.

## Competing interests

The authors declare that they have no competing interests.

## Authors' contributions

MMS, CSGC, PKM and HGS participated in the design of the study. MMS, PKM, PR, DFM and CE performed the data collection. MMS, LFSC, FYN performed the statistical analysis. MMS, DFM, SRM and HGS wrote the manuscript. All authors read and approved the final manuscript.
